# Rectal In Situ Thermosensitive Gel Loaded with Agomelatine-Silver Nanoparticles: Formulation and Characterization

**DOI:** 10.3390/gels12010051

**Published:** 2026-01-02

**Authors:** Marwa H. Abdallah, Mohamed S. Mohamed, Tamer M. Shehata, Wael A. Abdelhafez, Mahmoud M. A. Elsayed, Abd El hakim Ramadan, Islam Kamal, Abdulsalam M. Kassem, Mahmoud Elkot Mostafa, Ayman Salama, Reda A. Mahmoud, Ahmed A. El-Shenawy

**Affiliations:** 1 Department of Pharmaceutics, College of Pharmacy, University of Ha’il, Ha’il 81442, Saudi Arabia; 2 Department of Pharmaceutics and Pharmaceutical Technology, Faculty of Pharmacy, Al-Azhar University, Assiut 71524, Egypt; 3 Department of Pharmaceutical Sciences, College of Clinical Pharmacy, King Faisal University, Al-Ahsa 31982, Saudi Arabia; 4 Department of Pharmaceutics and Clinical Pharmacy, Faculty of Pharmacy, Sohag University, Sohag 82524, Egypt; mahmoudalmenshawy@pharm.sohag.edu.eg; 5 Department of Pharmaceutics, Faculty of Pharmacy, Port Said University, Port Said 42515, Egypt; 6 Department of Pharmaceutics and Pharmaceutical Technology, Faculty of Pharmacy (Boys), Al-Azhar University, Nasr City, Cairo 11651, Egypt; 7 Department of Pharmaceutical Technology, Faculty of Pharmacy, Menoufia National University, 70 km Cairo-Alexandria Agricultural Road, Menofia 32871, Egypt; 8 Department of Pharmaceutics and Industrial Pharmacy, Faculty of Pharmacy, New Valley University, Al-Kharga 72511, Egypt; 9 Department of Pharmaceutics, Faculty of Pharmacy, University of Tabuk, Tabuk 71491, Saudi Arabia; agrawan@ut.edu.sa; 10 Al-Azhar Centre of Nano Sciences and Applications, Al-Azhar University, Assiut 71524, Egypt; 11 Department of Pharmaceutics and Pharmaceutical Technology, Faculty of Pharmacy, Badr University, Assiut 71536, Egypt

**Keywords:** agomelatine, silver nanoparticles, green biosynthesis, thermosensitive gel, rectal drug delivery, enhanced bioavailability, sustained release

## Abstract

Agomelatine (AG) is a novel antidepressant characterized by distinct mechanism of action and minimal side effects. However, extensive first-pass hepatic metabolism limits its clinical efficacy after oral administration, leading to low bioavailability (<5%). To get around these restrictions, the current study set out to create and assess a rectal thermosensitive in situ gel using biosynthesized AG-silver nanoparticles (AG-AgNPs). AG-AgNPs were successfully synthesized with gum acacia as a stabilizing agent, using silver nitrate as a precursor, and ascorbic acid as a reducing agent. The in situ gel formulation was optimized using a 3^2^ factorial design, and then physicochemical, in vitro, and in vivo assessments were conducted. Nanoparticle formation was also evidenced by the appearance of a visible color change, UV-VIS, TEM, and XRD analysis techniques, which depicted spherical-shaped nanoparticles and a crystalline nature. The formulated optimized thermosensitive in situ gel showed good properties, which included drug content of 91.64%, gelation temperature of 26.63 °C, pH of 7.2, gel strength of 36.98 s, and sustained drug release of 80.24% in 6 h. The relative bioavailability in animal studies showed a remarkable increase in systemic availability with 277.5% relative bioavailability in comparison to an oral tablet formulation. In summary, results show that the AG-AgNP-loaded thermosensitive in situ gel could have potential use as a rectal delivery drug for bypassing first-pass effects and improving bioavailability for the drug Agomelatine.

## 1. Introduction

Agomelatine (AG) (N-[2-(7-methoxy-1-naphthyl) ethyl] acetamide) is a novel antidepressant distinguished by its unique mechanism of action, which involves acting as an agonist of melatonin receptors (MT1 and MT2) and an antagonist of the 5-hydroxytryptamine receptor (5-HT2C) [1,2]. This mechanism not only ensures comparable antidepressant efficacy but also offers advantages such as improved tolerability, fewer side effects, and enhanced sleep quality [3]. However, AG’s oral bioavailability is remarkably low (less than 5%), primarily due to extensive hepatic metabolism. Classified as a BCS Class II drug, AG is characterized by low aqueous solubility, high permeability, a short half-life (~1.5 h), and significant plasma protein binding (~90%) [4]. To address these limitations, advancements in nanoparticle (NP) technology offer promising solutions. Metallic nanoparticles (NPs), defined as particles with diameters below 100 nm, are gaining traction across various fields, including medicine and pharmaceuticals [5]. Among these, silver nanoparticles (AgNPs) have garnered particular interest due to their distinctive physical and chemical properties, biocompatibility, and applications in drug delivery systems as antimicrobial, anti-inflammatory, and anticancer agents. Conventionally, AgNPs are synthesized via chemical, physical, and photochemical methods, with chemical reduction being the most common approach [6]. However, the use of reactive chemical agents such as hydrazine and polyvinylpyrrolidone can pose environmental and biological toxicity concerns. To mitigate these drawbacks, green synthesis methods following the principles of green chemistry have emerged as an eco-friendly alternative [7]. These techniques employ non-toxic solvents, natural reducing agents, and biodegradable stabilizing agents to produce nanoparticles with high stability and uniform dispersion, thus minimizing adverse environmental impacts [8].

AgNPs have emerged as promising carriers for antidepressant drugs. They are offering several advantages, such as enhanced therapeutic efficacy, safety, and patient compliance [9]. The high surface area-to-volume ratio of AgNPs can enhance the dissolution rate of poorly soluble drugs, enhancing their aqueous solubility with better dispersion in the biological fluids [10]. Laghari et al., 2021 showed AgNPs improved the oral bioavailability of sertraline and reduced depressive symptoms in a rodent animal model via enhancing CNS targeting [11].

The rectal route of drug administration presents a compelling alternative for systemic drug delivery, offering advantages such as reduced enzymatic degradation, bypassing the hepatic first-pass effect, and improving drug absorption and patient compliance [12]. While traditional suppositories are commonly used, but they often suffer from drawbacks such as, discomfort, patient rejection, and limited bioavailability due to first-pass metabolism [13]. In contrast, thermosensitive mucoadhesive in situ gels, which remain liquid at ambient temperature and gelate upon administration, represent a more effective solution [14]. These gels prolong residence time at the site of absorption, enhance drug bioavailability, and provide better patient acceptability [15,16].

AG oral administration faces significant challenges due to its pharmacokinetic profile, making rectal delivery a promising alternative. AG undergoes extensive first-pass metabolism in the liver via CYP1A2, resulting in an oral bioavailability of ~5%, and food intake further reduces bioavailability by ~50% [17]. Additionally, oral intake of AG results in nausea, diarrhea, and abdominal pain [18]. The parenteral (intramuscular or intravenous) administration of AG is impractical for chronic depression management due to pain, infection risk, and required healthcare supervision, along with transient high plasma drug levels with the risk of adverse effects [19].

In this context, the current study focuses on the development and evaluation of a novel rectal in situ gel formulation of AG-loaded with AgNPs. The study aims to overcome the challenges associated with AG’s oral delivery by enhancing its bioavailability and absorption via the rectal route. The green-synthesized AgNPs were thoroughly characterized and incorporated into a thermosensitive in situ gel base. Key physicochemical parameters, including gelation temperature, gel strength, and in vitro AG release, were optimized. Additionally, the in vivo pharmacokinetics of the developed formulation were assessed to confirm its potential as a safe, effective, and patient-friendly alternative for depression treatment.

## 2. Results and Discussion

### 2.1. UV-Vis Spectral Analysis

**Figure 1:**
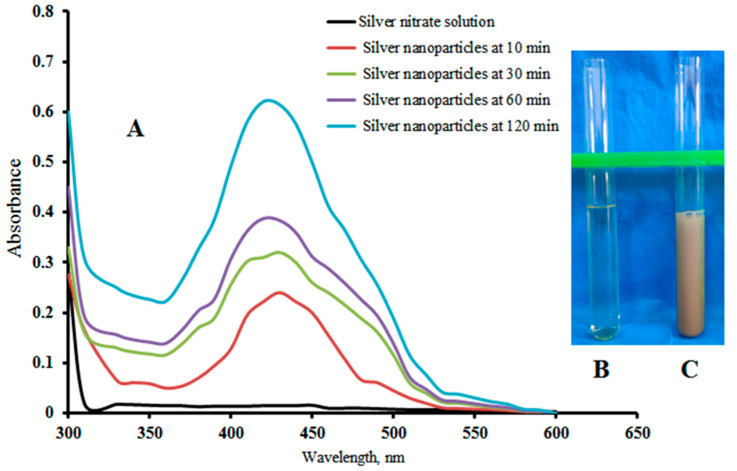
(**A**) UV Spectrophotometry of AgNPs at different time intervals, (**B**) before addition of silver nitrate, and (**C**) after addition of silver nitrate (2 h).

UV-Vis spectroscopy is one of the most popular methods for structural characterization of silver nanoparticles. The UV-Vis absorption spectra of the synthesized AgNPs were obtained against deionized water to investigate the synthesis and stability of the AgNPs [20]. The UV-Vis absorption spectra of the AgNPs at various time intervals are recorded and shown in Figure 1A. The AgNPs synthesis during the reduction process is indicated by the color change in the reaction solution from colorless to dark brown, which can be visually observed. AgNPs have free electrons, which produce a surface plasmon resonance absorption peak. The colors of the silver nitrate and AgNPs solution (after 2 h) are shown in Figure 1B,C, respectively. The evolution of the surface plasmon absorbance peaks of silver nanoparticles during the first two hours is shown in Figure 1A. The solution color was changed within seconds to pale yellow, and then to dark brown, due to the plasmon formation at the colloid surface, indicating the synthesis of AgNPs [21].

It can be observed that the absorbance peak maxima of AgNPs are in the range of 400–425 nm due to the surface plasmon resonance of silver nanoparticles. The conduction electrons undergo oscillation due to the strong interaction of light with the synthesized AgNPs. The reaction between Ag+ and the reducing material was followed for 120 min, and UV-Vis measurements were conducted at 10, 30, 60, and 120 min. Figure 1A shows the UV-Vis spectra of AgNPs as a function of time. The silver reduction, as well as nucleation and growing size of the synthesized silver nanoparticles, increase from 10 min to 120 min. The reaction time resulted in a gradual increase in UV-absorbance peaks. The color intensity of the obtained system changes from light yellow to dark brown at the end of the reaction because of the rise in AgNPs and the exhibited particle aggregation [22].

### 2.2. DL%

Surface adsorption is the main mechanism of DL on AgNPs. During the trials, the increase in drug concentration above 1 mg/mL does not affect DL. When 0.5 mg/mL was added, the DL% was 69.4%. When 1 mg/mL was added, the DL% was 89.1%. When the drug concentration was further increased, the DL% was decreased, which could be due to the saturation of the AgNPs surface [23].

### 2.3. Morphology of the Synthesized AgNPs

**Figure 2:**
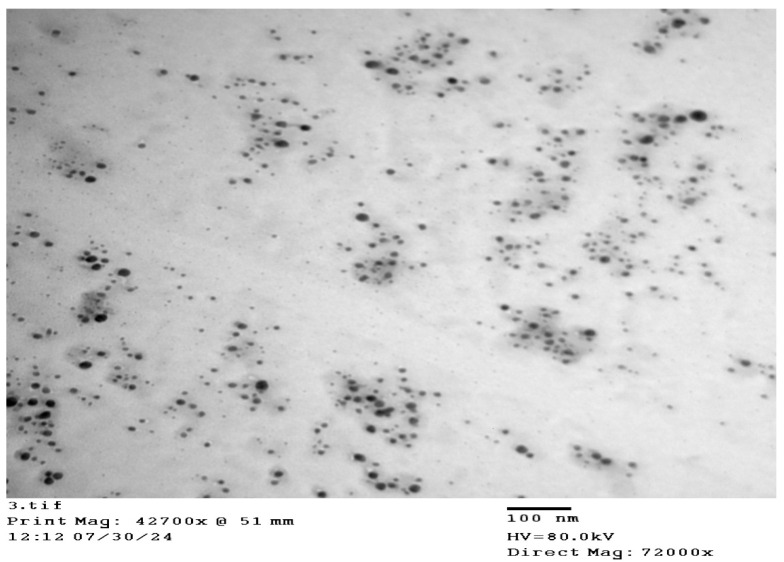
TEM image of AgNPs synthesized with 0.5%, *w*/*v* gum acacia and 1 mM silver nitrate.

Figure 2 shows the TEM image of the synthesized AgNPs. The TEM image for the investigated AgNPs samples shows the spherical shape of the synthesized particles, nearly monodispersed in nature, in addition to aggregated particles with larger sizes [24]. The particle size ranged from 26 to 49 nm, and the average particle size obtained from the corresponding diameter distribution was about 42.14 ± 1.6 nm.

### 2.4. Particle Size and Zeta Potential

**Figure 3:**
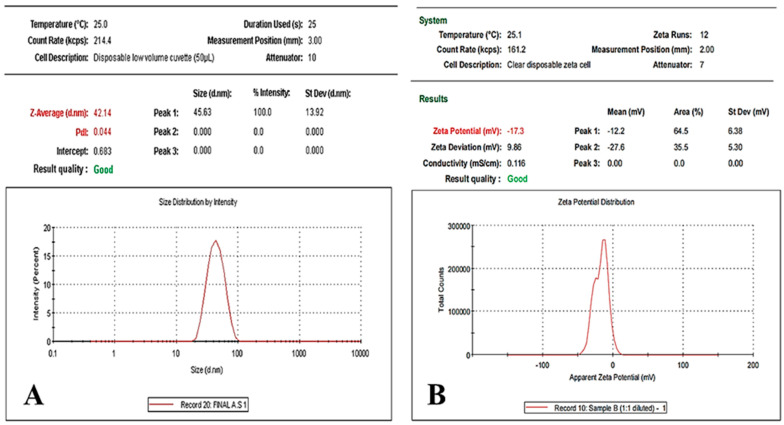
(**A**) Particle size and (**B**) Zeta potential of the synthesized AG-loaded AgNPs.

The results obtained showed that the average particle size of the synthesized AG-loaded AgNPs is 42.14 nm with a polydispersity index (PDI) of 0.044 (Figure 3A). Another important characterization parameter of AgNPs is the determination of nanoparticle surface charge (zeta potential). The magnitude of the zeta potential signifies the degree of electrostatic repulsion between similarly charged neighboring particles in a dispersion, which, in turn, dictates the stability of colloidal dispersions [25]. As illustrated in Figure 3B, the synthesized AG-loaded AgNPs had a zeta potential of −17.3 ± 1.12 mV, indicating their stability.

### 2.5. PXRD

**Figure 4:**
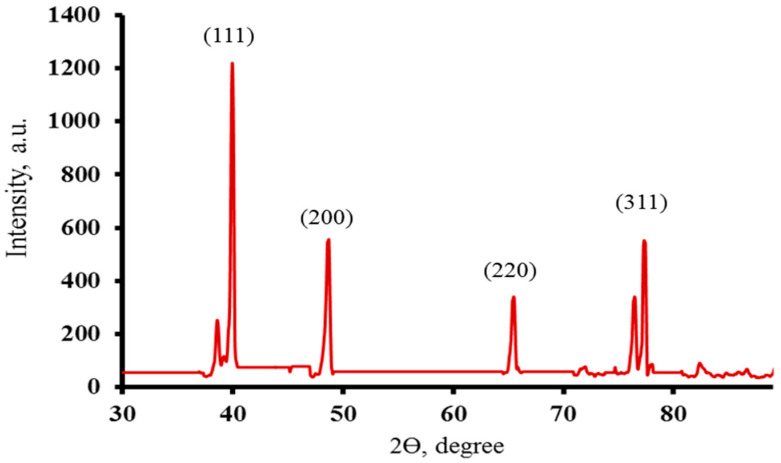
X-ray diffraction pattern of the synthesized AG-loaded with AgNPs.

The crystal structure of the produced AG-loaded AgNPs was defined and highlighted using PXRD analysis. The crystalline character of AG-loaded AgNPs was confirmed by the obtained PXRD diffractogram (Figure 4). As indicated in Figure 4, the XRD diffractogram revealed four main peaks at 2θ values of 40.06°, 46.18°, 65.32°, and 77.26°, which may be caused by silver-metal in the AG-loaded AgNPs and correspond to (hkl) values of (111), (200), (220), and (311) reflection planes of the face-centered cubic structure silver. The diffractogram shows more peaks, which could be the result of unreduced silver nitrate remaining in the sample in trace amounts. The results obtained are consistent with findings published by other researchers [26].

### 2.6. FT-IR Spectroscopy Analysis

**Figure 5:**
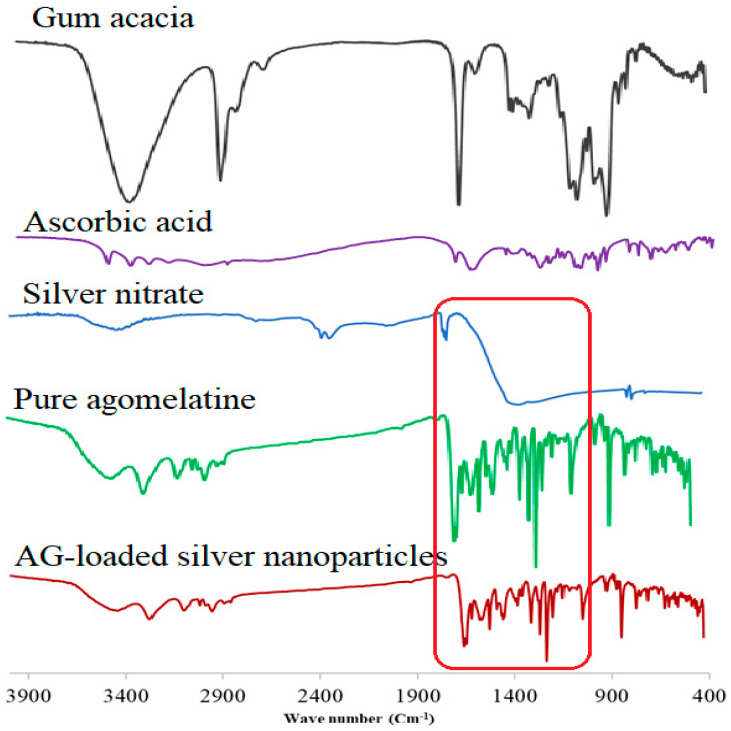
FTIR spectra of pure agomelatine, unprocessed silver nitrate, gum acacia, ascorbic acid, and the prepared silver nanoparticles.

The FT-IR spectrum of AG (Figure 5) showed significant bands belonging to molecular groups such as phenyl, ether bond, secondary amine, and carbonyl group. The absorption band due to stretching vibration of N−H, =CH (phenyl), and C=C groups at 3246.21, 3074.63, 1550.74 cm^−1^, respectively [27]. Additionally, the spectrum showed characteristic bands for –CH_2_ aliphatic chain asymmetrical stretching vibration, C=O carbonyl stretching vibration, and C−O−C ether bond asymmetrical stretching vibration at 2940.66, 1640.00, and 1216.54 cm^−1^, respectively [28]. The FT-IR spectra of AgNO_3_ showed bands at 3452.62, 2355.16, and 1753.02 cm^−1^ with a broad, high-intensity band at 1378.38 cm^−1^ due to the stretching vibration of N=O [29]. The FT-IR spectra of Gum acacia have shown characteristic bands at 3423.83, 2927.49, and 1612.24 cm^−1^, due to the aliphatic –OH, aliphatic –CH_2_, and asymmetric and symmetric stretching of COO− group, respectively [30]. The band at 1426.43 cm^−1^ is due to the −OH group bending of the acid group [31]. The FT-IR spectrum of unprocessed ascorbic acid showed a stretching vibration band of C=C at 1674.14 cm^−1,^ and the enol-hydroxyl group was responsible for the band observed at 1321.00 cm^−1^ [32]. For the synthesized AgNPs, the FT-IR spectrum showed some shifting of the bands; band intensity decreased/increased, and disappeared, as shown in Figure 5. The shifted bands revealed that the responsible functional groups were involved in the reduction and stabilization process [33]. The shifting in the shape and peak positions of the –OH, −COO− groups belonged to gum acacia at 1446.89 and 1638.82 cm^−1^, respectively, observed because of the contribution toward the reduction and stabilization process [34] (Figure 5). The obtained FTIR results revealed that silver nanoparticles interacted with the different methoxy, amide, and naphthalene groups of AG, assuming that these interactions are responsible for a great manner of the stability of colloidal solutions [35]. Additionally, the absence of characteristic nitrate bands of AgNO_3_ from the spectrum of the synthesized silver nanoparticles confirms the complete reduction in Ag^+^ ions and the removal of residual nitrate species (Figure 5).

### 2.7. Characterization of the Fabricated In Situ Rectal Gel Formulations

#### 2.7.1. Impact of Independent Variables on GT

**Figure 6:**
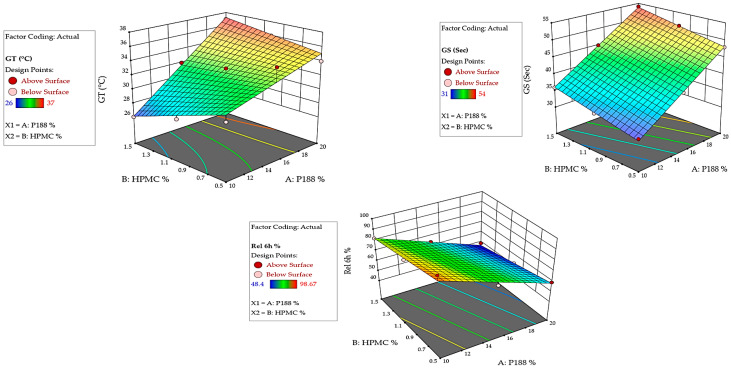
Three-dimensional response surface plots estimating the effect of independent variables on the gelation temperature, the gel strength, and the cumulative % AG released after 6 h.

**Figure 7:**
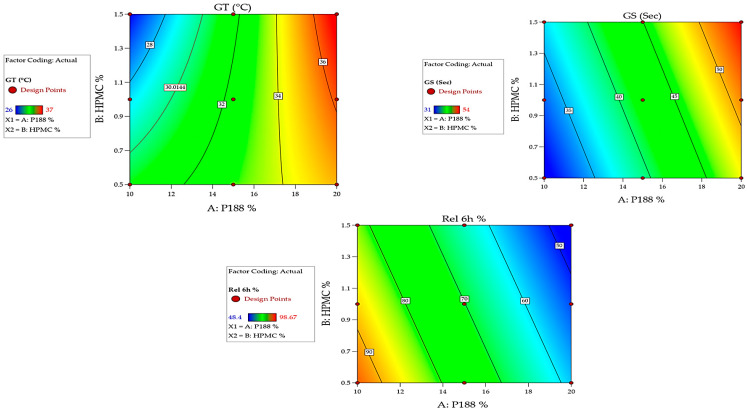
Contour plots estimating the effect of independent variables on the GT, the GS, and the cumulative % AG released after 6 h.

The GT is a critical parameter for in situ rectal gels, as it determines the temperature at which the gel transitions from a liquid to a gel state [36]. The data shows that GT increases at P188 percent higher. Across all investigated in situ gel formulations, as the P188 percent increases from 10 to 20%, *w*/*v*, there is a noticeable increase in the GT (from 26 to 37 °C). This suggests that P188 plays a significant role in raising the GT, making the gel more stable at higher temperatures [37]. This trend is consistent across varying levels of HPMC K15M percent. The results obtained showed that the increase in HPMC K15M percent from 0.5 to 1.5%, *w*/*v* results in a slight decrease in GT within the same P188 percent formulation. This effect is less pronounced than that of P188, indicating that while HPMC K15M does influence GT, its effect is secondary to that of P188 [38]. The significant effect of P188 on GT is corroborated by the ANOVA results, where the P188 term (A) shows a highly significant F-value (Figure 6 and Figure 7 and Table 1), indicating a strong influence on GT. The following equation can be applied to explain the effect of P188 and HPMC K15M percent on the GT.

GT (°C) = +32.6667 + 0.066667 P188% − 11.83333 HPMC% + 0.700 P188% HPMC%

#### 2.7.2. Impact of Independent Variables on GS

The GS is a measure of the mechanical properties of the in situ gel, which are crucial for ensuring that the gel remains intact during administration [39]. The data shows a clear increase in GS as the percentage of both P188 and HPMC K15M is increased. For example, GS rises from 31 s in F1 (10%, *w*/*v* P188 and 0.5%, *w*/*v* HPMC K15M) to 54 s in F9 (20%, *w*/*v* P188 and 1.5%, *w*/*v* HPMC K15M). This suggests a synergistic effect where both P188 and HPMC contribute to a more robust in situ gel matrix. The impact of P188 is more pronounced at a higher percentage of HPMC K15M. The increase in GS is more significant when both P188 and HPMC K15M are at their highest percent (F9). This indicates that the interaction between P188 and HPMC K15M is critical in enhancing the mechanical properties of the prepared in situ gel [15]. The interaction term (AB) in the ANOVA table shows significance, supporting the observation that the combined effect of P188 and HPMC on GS is non-additive and noteworthy (Figure 6 and Figure 7 and Table 1). The following equation can be applied to explain the effect of P188 and HPMC K15M percent on the GS.

GS (s) = 9.94444 + 1.76667 P188% + 5.66667 HPMC%

#### 2.7.3. Syringeability

All the prepared in situ gel formulations passed the syringeability test as the investigated in situ gel samples were expelled quite easily from the 22-gauge needle syringe (Table 2). Syringeability also significantly depended upon the percentage of P188 and HPMC K15M. The obtained results revealed that as the polymer percentage increased, the viscosity of the fabricated in situ gel formulations also increased, which required a high force to expel the gel from the 22-gauge needle syringe [40].

#### 2.7.4. Spreadability

Spreadability was expressed at the area (cm^2^) in which the investigated in situ gel readily spreads. The obtained results showed that all the formulated in situ gel formulations with various P188 and HPMC K15M percentages were found to be spreadable, and the spreading extent was inversely proportional to the polymer percentage (spreading area increases with a decrease in the percentage of P188 and HPMC K15M and vice versa) [41]. The diameter of the spreading area for the investigated in situ gel formulations was found in the range of 5.13 ± 0.24 to 7.46 ± 0.31 cm^2^, as listed in Table 2.

#### 2.7.5. Rheology

The drug release rate, distribution, in vivo retention time, and in vivo behavior could be affected by the viscosity of the rectal in situ gel [41]. The viscosity of the fabricated in situ gel formulations is shown in Table 2. At 25 °C, all the formulations were in liquid form with low viscosity and exhibited a Newtonian behavior, ensuring the homogeneous spreading on the rectal mucosa. At 37 °C, the results obtained revealed that as the concentration of the gelling agents increased, the apparent viscosity increased (pseudoplastic flow) [42]. The viscosity of the P188 solution increases as the temperature increases, which results in the system gelation due to the aggregation of P188 molecules into spherical micelles [43]. From the obtained results, it was found that as the percent of HPMC K15M increased, the viscosity of the fabricated in situ gel formulations increased, which could be attributed to the water-absorbing capacity of HPMC K15M (hydrophilic polymer), which has increased viscosity [15].

#### 2.7.6. Drug Content and pH Evaluation

The prepared in situ gel formulations were elegant, white, homogenous, smooth in texture, and free of air bubbles and lumps. The pH value of the investigated in situ gel was found to range between 5.9 ± 0.43 and 7.3 ± 0.31, so it is considered rectally tolerable and expected to be free of any pH-related harmful actions [41] (Table 2). Further, the AG content percentage of the fabricated in situ gel formulations ranged from 89.67 ± 2.43 to 96.55 ± 1.27%, indicating good formulation homogeneity (Table 2).

#### 2.7.7. Impact of Independent Variables on the Cumulative Percent Released at 6 h

**Figure 8:**
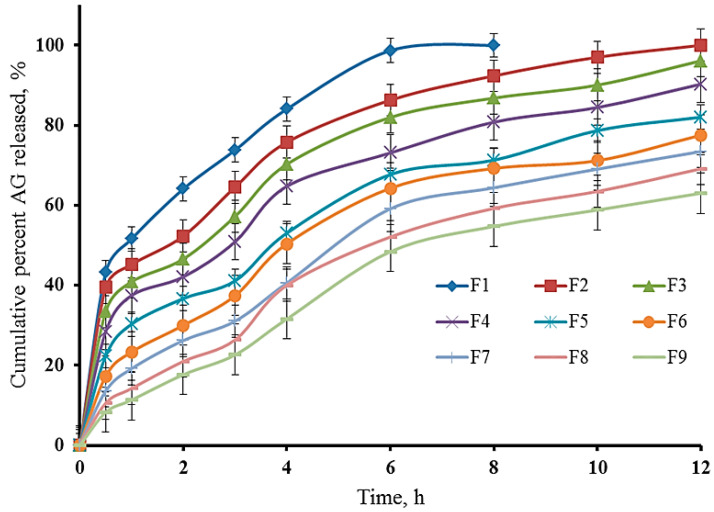
In vitro release profile of AG from various rectal in situ gel formulations (data expressed as the mean ± SD, n = 3). The various formulations are coded with F1, F2, F3, … and F9.

Cumulative drug release is a key performance indicator for the in situ gels, as it reflects the ability of the formulated in situ gel to deliver the drug over time [44]. The results obtained showed that the cumulative percentage amount of AG released at 6 h decreases with increasing P188 and HPMC K15M percent. The release profile of AG shows a decreasing trend as both P188 and HPMC K15M percent increase (Figure 8). For instance, the AG release drops from 98.67% (F1) to 48.4% (F9). This suggests that a higher percentage of P188 and HPMC K15M results in a denser in situ gel matrix, which in turn slows down drug diffusion [15]. The results also revealed an inverse relationship between the drug release and GS. As GS increases, drug release decreases. This inverse relationship highlights the trade-off between mechanical robustness and drug release efficiency [45]. Formulations with higher GS (e.g., F9) retain the drug for longer periods, which could be beneficial for sustained release applications but may not be suitable for immediate release needs [46]. This trend is supported by the lower *p*-value associated with the P188 percent in the ANOVA results, indicating its strong influence on drug release (Figure 6 and Figure 7 and Table 1). The following equation can be applied to explain the effect of P188 and HPMC K15M percent on the cumulative percent AG amount released at 6 h.

Release at 6 h (%) = 135.91722 − 3.57700 P188% − 12.08333 HPMC%

The factorial design analysis indicates that P188 percent has the most significant effect on GT, GS, and drug release, with HPMC K15M also playing a vital role, especially in enhancing GS. The interaction between P188 and HPMC K15M is crucial in determining the final properties of the gel. The kinetic analysis illustrated that the release of AG from the formulated rectal in situ gel formulations fit best with Higuchi’s diffusion model, which has the highest R^2^ values, Table 3.

### 2.8. Optimization of Rectal In Situ Gel Formulations

**Figure 9:**
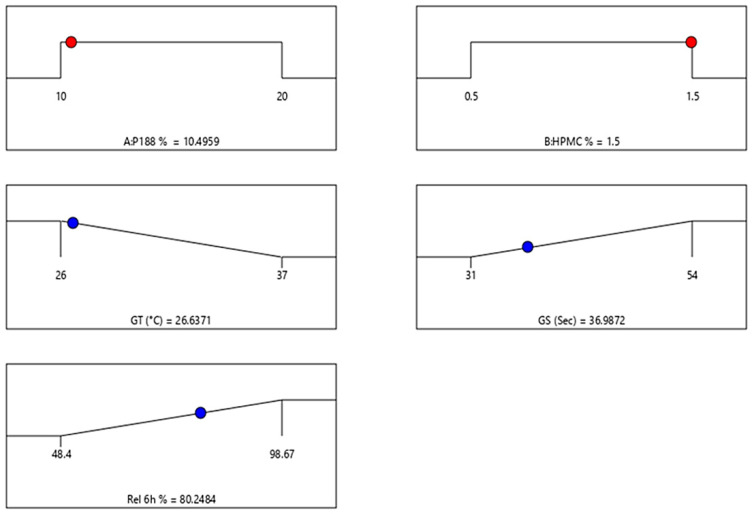
Composition and the dependent variables of the optimized rectal in situ gel formulation.

The preparation of the optimized in situ gel formula and its subsequent evaluation confirm that the formulation meets the intended criteria for an effective in situ rectal gel. The selected percent of P188 (10.5%, *w*/*v*) and HPMC K15M (1.5%, *w*/*v*) provides a favorable GT, adequate GS, and a robust drug release profile, making the optimized in situ gel formulation suitable for practical use (Figure 9). The optimization process highlights the importance of carefully balancing these factors to achieve a formulation that is not only effective but also patient-friendly. The optimized in situ gel formula, with its measured values of GT (26.63 ± 2.11 °C), GS (36.98 ± 1.25 s), and release after 6 h % (80.24 ± 3.68%), offers a promising solution for the delivery of AG-AgNPs via the rectal route, combining efficacy with ease of use. The interaction between HPMC K15M and P188 in the optimized formula ensures a synergistic effect, where HPMC K15M provides the necessary structural integrity, and P188 ensures appropriate GT and drug release dynamics. This interaction was the key to achieving the desired balance in the optimized formula. The GS of 36.98 s in the optimized formula indicates a formulation that can adhere well to the rectal mucosa, maintaining its position for extended periods to allow for sustained drug release. This adhesion is critical for ensuring that the drug remains at the site of action. The optimized GS strikes a balance that ensures the gel is strong enough to remain intact during application but not so strong that it causes discomfort. This balance is essential for rectal in situ gel formulations, where patient comfort is paramount.

### 2.9. Histopathology Study

**Figure 10:**
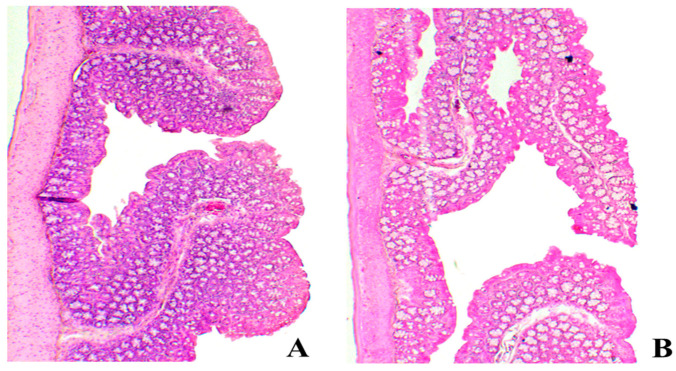
Histopathology photomicrographs of (**A**) untreated rectal mucosa and (**B**) rectal mucosa treated with optimized in situ gel formula.

To investigate the safety of the optimized in situ gel formula, rectal mucosal damage/irritation was investigated by the histological study of the rabbit rectal tissues after the administration of the gel formula [13]. Figure 10 shows the morphology of the control rabbits’ rectal mucosa (group I) (Figure 10A) and after the rectal administration of the optimized in situ gel formula (group II) (Figure 10B). Group I showed normal mucosal structure. Group II showed no evidence of significant epithelial necrosis or hemorrhage. It showed only a few eosinophils infiltrating the rectal mucosa. The obtained results are in good accordance with Yuan et al., 2012 reported previously that Na-Alg, HPMC K15M, and poloxamers caused no damage to rectal mucous membranes [16].

### 2.10. Retention In Vivo Test

One of the important rectal in situ gel characteristics is their ability to exhibit mucoadhesion. The results obtained showed that after giving the optimized in situ gel formulation to the rabbits, the leakage rate of all animals was 16 ± 1.4%. The leakage due to defecation was also considered in the leakage rate. This indicated that the mucoadhesive force of the investigated rectal in situ gel was strong enough to exhibit good contact between the fabricated in situ gel and the rabbit’s mucosal tissues. The mucoadhesion mechanism could be attributed to the hydrogen bonding between the fabricated gel (carboxyl groups of Na-Alg) and the oligosaccharide chains of the animal rectal mucous lining [47].

### 2.11. In Vivo Assessment

**Figure 11:**
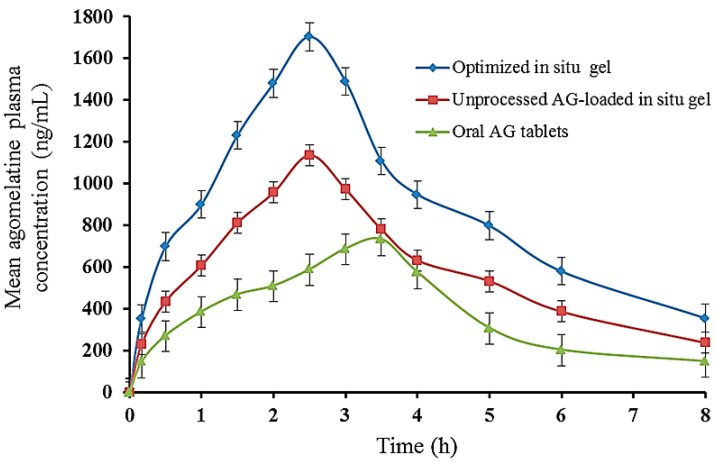
The rabbit plasma concentration-time curve after a single-dose administration of different agomelatine dosage forms (The results are expressed as the mean ± SD, n = 3).

Pharmacokinetic studies were performed after the administration of AG by both the oral route (AG tablets) and rectal route (the optimized in situ gel and unprocessed AG-loaded in situ gel formulations). The plasma level-time curves of the rabbits are shown in Figure 11, and the pharmacokinetic parameters of the obtained data are listed in Table 4. It was noticed that both the in situ gel formulations reached their maximum plasma concentration (t_max_) more rapidly (after 2.5 ± 0.251 h) than the oral AG tablets (3.5 ± 0.158 h). The obtained t_max_ reflected the changeability in AG absorption among different administered formulations. Regarding the C_max_ values, the rabbit plasma concentrations of AG in the case of the optimized in situ gel formulation were higher than the corresponding C_max_ of the unprocessed AG-loaded in situ gel and the oral AG tablets. Higher plasma concentrations in the case of optimized in situ gel formulation could be attributed to the dispersibility and bioadhesive force variation. In the case of the optimized in situ gel, the dispersion was more pronounced in the rectal fluids as AG was introduced in a soluble AgNPs form [48]. In the case of the unprocessed AG-loaded in situ gel and AG oral tablets, pure AG was poorly soluble. The t_1/2ab_ of the optimized in situ gel formula (1.576 ± 0.376 h) was higher than the unprocessed AG in situ gel formula (1.37273 ± 0.264 h) and oral AG tablets (0.527 ± 0.251 h) (*p* < 0.05), which might be due to the sustained release of AG, owing to the bioadhesive in situ gel matrix of poloxamer, HPMC K15M, and Na-Alg created at animal body temperature [13]. A longer t_1/2el_ was observed for the optimized in situ gel and the unprocessed AG-loaded in situ gel formulations when compared to the oral AG tablets, which could assist in decreasing the frequency of the drug dosing. In turn, this also reflected a higher MRT of the optimized in situ gel formula (5.138 ± 0.035 h) and unprocessed AG-loaded in situ gel (4.148 ± 0.026 h) when compared to the oral AG tablets (3.049 ± 0.0211 h). The optimized in situ gel formula also provided a significantly (*p* < 0.05) higher AUC_0–8_ (7068.262 ± 72.98 ng × h/mL) than the unprocessed AG-loaded in situ gel (4692.648 ± 81.24 ng × h/mL) and oral AG tablets (3039.735 ± 35.46 ng × h/mL). The results showed enhanced AG bioavailability by employing the in situ rectal gel formulation compared to the oral AG tablets. The rectal route of administration can generally protect AG from the extensive first-pass metabolism followed by oral administration. The prefabrication of the AG-AgNPs enhanced AG solubility. Additionally, the mucoadhesion effect of P188 and Na-Alg prevented the drug from traveling into the upper haemorrhoidal vein and improved lower rectum residence. Drug absorption via the lower haemorrhoidal vein, through which the drug enters directly into the systemic circulation, could boost AG bioavailability. The RB of the optimized in situ gel and unprocessed AG-loaded in situ gel formulations were calculated to be 277.5% and 182.9%, respectively, compared with the commercially available AG oral tablet. The results obtained implied that the AG-AgNPs-loaded in situ gel could effectively deliver AG rectally.

### 2.12. Accelerated Stability Study

The stored optimized rectal in situ gel formulation was evaluated for various predetermined parameters after exposure to different storage conditions (25 ± 0.5 °C and 40 ± 1 °C/relative humidity 75% for 3 months) [49]. The results obtained revealed that there were no significant differences in the evaluated parameters from initial to after accelerated stability studies, as listed in Table 5, and all of the results were found to be within acceptable limits after the stability investigations for the optimized rectal in situ gel formulation.

## 3. Conclusions

This study introduces a novel in situ gel system for rectal delivery of AG-AgNPs, providing a breakthrough in enhancing AG bioavailability. Utilizing a green synthesis approach, stable AG-AgNPs were fabricated with a uniform size of 42.14 nm and a zeta potential of −17.3 mV, leveraging gum acacia and ascorbic acid as eco-friendly reducing and stabilizing agents. Comprehensive characterization confirmed their stability and suitability for advanced drug delivery. The optimized gel formulation—comprising P188 (10.5%, *w*/*v*), HPMC K15M (1.5%, *w*/*v*), and Na-Alg (0.5%, *w*/*v*)—exhibited desirable properties, including rapid gelation (36.98 s), optimal gelation temperature (26.63 °C), and sustained drug release (80.24% over 6 h). Stability and safety testing further validated its robustness for rectal administration. Pharmacokinetic analysis revealed a remarkable 2.775-fold enhancement in AG bioavailability compared to oral tablets, effectively addressing challenges of low bioavailability and first-pass metabolism. This innovative system not only enhances therapeutic efficiency but also opens new avenues for the rectal delivery of challenging drugs, making it a compelling alternative for depression treatment and beyond. Researchers are encouraged to explore its potential for broader applications in drug delivery science.

## 4. Materials and Methods

### 4.1. Materials

Agomelatine (AG) (molecular weight, 243.301 gm/mol) was kindly provided by Mash Premiere Pharmaceuticals, Cairo, Egypt. Methyl alcohol was purchased from El Nasr Pharmaceutical Chemicals Co., Cairo, Egypt. Potassium dihydrogen orthophosphate and disodium hydrogen orthophosphate were obtained from NICE Chemicals (p) LTD, Kerala, India. Silver nitrate was obtained from Sigma Aldrich, Darmstadt, Germany. Poloxamer 188 (P188) was obtained as a free sample from Novartis Pharma S.A.E., Cairo, Egypt. Hydroxypropyl methylcellulose (HPMC, K15M), sodium alginate, and Gum acacia were purchased from Aldrich Chem. Co., St. Louis, MO, USA. Ascorbic acid was purchased from Sigma-Aldrich (St. Louis, MO, USA). Dialysis cellulose membrane with a molecular weight cut off of 12,000 Da was obtained from Sigma-Aldrich Co., St. Louis, MO, USA. HPLC-graded methyl alcohol was purchased from Merck Schuchardt, Hohenbrunn, Germany. All other solvents and reagents used were of analytical grade.

### 4.2. Animals

For all in vivo studies, male albino rabbits weighing 1.8–2.25 kg were used. All animals were habituated at ambient temperatures with free access to diet and water. The animal experimental procedures were approved by the Ethical Committee of Al-Azhar University, Faculty of Pharmacy, Assiut branch, Egypt, according to the animal certificate no. AZ-AS/PH-REC/60/2024 on 27 July 2024.

### 4.3. HPLC Analysis of AG

For the AG in vivo assessment, the AG content was estimated by the high- performance liquid chromatography (HPLC) technique. The chromatographic conditions utilized for the analysis are given in Table 6. Before the analysis procedures, the mobile phase was freshly prepared, filtered, and degassed. A linear correlation was acquired between the calculated peak area and AG concentration. The linear equation was y = 438.54x − 4518.2 (R^2^ = 0.997), where x is the concentration and y is the peak area. The assay was linear in the concentration of 1–150 µg/mL. The detection limit was 0.5 µg/mL. All experiments were carried out in triplicate, and the mean ± standard deviation (SD) was considered.

### 4.4. Green Synthesis of Silver Nanoparticles

Gum acacia (0.5 gm) and silver nitrate (0.4 gm) amounts were dissolved in deionized water (100 mL) under continuous stirring at 1500 rpm (MSH-ORO, Massachusetts, USA), followed by the addition of ascorbic acid (10 mg/mL) with further stirring for 30 min. The obtained mixture turned dark brown immediately, indicating the reduction in Ag+ and the subsequent AgNPs formation [50]. For further reduction in AgNPs, the mixture was subjected to ultrasonication by an ultrasonic processor (GE130; probe CV18, Sonics & Materials, Inc., Newtown, CT, USA) for 10 min. To the previous mixture, the dissolved amount of AG in (1 mg/mL) methyl alcohol was added drop by drop [51]. The obtained AgNPs were stored in dark conditions for further characterization by UV-Vis spectrophotometry, TEM, particle size, and zeta potential. To obtain dry silver nanoparticles, colloidal AgNPs were centrifuged at 15,000 rpm for 1 h at 4 °C using a cooling centrifuge (Biofuge^®^ primo, Frankfurt, Germany), and the supernatant was removed; the precipitated AgNPs were washed twice with distilled water before lyophilization for 72 h (Freeze dryer, Acculab, FD55-10S, New York, NY, USA) [52]. The lyophilized AgNPs obtained were stored in a desiccator until further characterization by Fourier-Transform Infrared spectroscopy and X-ray diffraction pattern.

### 4.5. Characterization of AgNPs

#### 4.5.1. UV-Vis Spectrophotometry

UV-Vis spectra of AgNPs were monitored in the wavelength range from 200 to 800 nm using a UV-Vis spectrophotometer (Shimadzu, model UV 1601 PC, Kyoto, Japan). An intense absorption peak in the UV-Vis spectra was found due to the strong surface plasmon resonance, indicating the formation of AgNPs [53].

#### 4.5.2. Drug-Loading (DL)

The loading of AG in the synthesized AgNPs was determined utilizing the supernatant obtained in Section 2.4. The free AG was determined spectrophotometrically at λ_max_ of 230 nm. The DL percentage was calculated according to the following equation [54].

% DL = AG_T_ − AG_F_/Weight of nanoparticles × 100

where AG_T_ is the AG amount used during the synthesis of the nanoparticles, and AG_F_ is the free AG in the supernatant.

#### 4.5.3. Morphology of the Synthesized AgNPs

The morphology of the synthesized AgNPs was investigated using transmission electron microscopy (TEM) (JEM-100 CX 11, Electron microscope, Jeol, Tokyo, Japan). A drop of the colloidal mixture was diluted with distilled water, sonicated for 10 min, and applied to a carbon-coated copper grid, then allowed to dry at room temperature [55]. The sample investigated was then examined and imaged by TEM at 80 kV.

#### 4.5.4. Particle Size and Zeta Potential

The mean particle size, polydispersity index (PDI), and zeta potential of the synthesized AgNPs were determined by dynamic light scattering (DLS) on Malvern Zetasizer, ZEN 1690, Nano-S90 (Malvern Instruments Limited, Worcestershire, UK) at room temperature at a scattering angle of 90 ° after appropriate dilution with distilled water [56,57,58]. The measuring procedure was conducted in a triplicate manner at room temperature.

#### 4.5.5. Powder X-Ray Diffraction (PXRD)

PXRD pattern of the synthesized AgNPs was obtained using a powder diffractometer (Philips 1710 diffractometer, Malvern Panalytical GmbH, Kassel, Germany) with Kα radiation, which operated at a voltage of 40 kV and a current of 30 mA in the 2θ range from 20 to 90° [59].

#### 4.5.6. Fourier-Transform Infrared (FT-IR) Spectroscopy Analysis

The chemical interaction between the functional group responsible for the reduction of silver ions to AgNPs was investigated by FT-IR spectroscopy analysis. The investigated samples were analyzed by a Fourier-Transform infrared spectrophotometer (IR-476-Shimadzu, Kyoto, Japan). Spectra were recorded in the transmission mode of 4000–400 cm^−1^ [60,61].

### 4.6. Appraisal and Optimization of AG-AgNPs-Loaded In Situ Rectal Gel

By 3^2^ full Factorial-design, nine formulations of in situ gel were designed by employing the software of Statgraphics Centurion, version XVII. Two independent variables, namely P188 percent (A), and HPMC K15M percent (B), were selected. However, gelation temperature (GT), gel strength (GS), and in vitro cumulative percent drug released at 6 h were selected as dependent responses. The proposed design is listed in Table 7. For the preparation of AG-AgNPs, thermosensitive in situ gel, P188, and HPMC K15M with various percentages were used. Sodium alginate (Na-Alg) was added to a constant percentage (0.5%, *w*/*v*). The previously reported cold method by Yuan et al., 2012 was slightly modified to fabricate an in situ gel [16]. Briefly, the accurately weighed amounts of mucoadhesive polymers (HPMC K15M and Na-Alg) were sprinkled onto the colloidal AG-AgNPs (20 mL) with continuous stirring till the mixture was totally dissolved. Afterward, the specified P188 amount was added portion-by-portion under continuous stirring, and then the obtained system was stored at 8 °C until needed. The concentration of AG used was 5 mg/5 mL. An unprocessed AG-loaded in situ gel formula was prepared for comparison with the optimized formula. Compositions of the prepared various in situ gel formulations are demonstrated in Table 7.

#### 4.6.1. Gelation Temperature (GT) Measurement

The GT (T_sol-gel_) of the fabricated in situ gel formulae was investigated according to the reported procedure by Liu et al., 2017 [62]. Briefly, 15 g of the investigated in situ gel formulation was charged into a transparent glass vial of a suitable size. The vial was then placed in a thermostatically controlled water bath, and the temperature was adjusted to 20 ± 0.5 °C with a thermosensor immersed in the vial content. The temperature was raised at a rate of 1 °C/min under constant stirring (100 rpm) with a magnetic bar. When the magnetic bar stopped rotating due to the gelation, the recorded temperature on the thermosensor was considered as GT.

#### 4.6.2. Measurement of Gel Strength (GS)

The GS of the fabricated in situ gel formulations was investigated by the procedure reported by El-Kamel et al., 2006 with some modifications [63]. Briefly, 50 mL of the investigated in situ gel formulation was charged into a 100 mL graduated transparent cylinder, and the cylinder was kept for 30 min in a water bath, and the temperature was adjusted to 36 ± 0.5 °C. A metal disk of a diameter of 3 cm weighing 35 gm was then placed on the surface of the investigated in situ gel formulation. GS was determined by the time required by the metal disk to penetrate up to 5 cm into the investigated in situ gel formulation. All the experiments were conducted in triplicate, and the mean ± SD was recorded.

#### 4.6.3. Syringeability Test

Syringeability is the ability of the fabricated in situ gel formulations to flow easily through the desired gauge needle. The experiment was conducted using a procedure reported by Mihir R. et al., 2021 [64]. The sample cold in situ gel was charged in a 5 mL plastic syringe equipped with a 22-gauge needle. The samples that were successfully passed from the syringe were considered passed.

#### 4.6.4. Spreadability Test

The investigated in situ gel sample (2 gm) was placed at the center of a clean, dry glass plate with a dimension of 10 × 10 cm, then it was covered with a glass plate with the same dimensions. A constant weight (500 gm) was carefully placed on the upper glass plate. After one minute, the weight was removed, and the diameter of the spread gel area was recorded [65,66].

#### 4.6.5. Rheological Study

The rheological properties of the prepared in situ gel formulations were determined using a Brookfield viscometer, DV-II Pro, AMETEK Brookfield, Middleboro, Massachusetts, USA, equipped with spindle no. 62. A 50 mL sample of each in situ gel formulation was placed in a glass container. Primarily, viscosity measurement was recorded at a speed of 50 rpm, and the samples were first investigated at 25 ± 1 °C, then the temperature was elevated to reach 37 ± 0.5 °C, and the viscosity was measured at the new temperature [67,68]. All measurements were conducted in a triplicate manner, and the mean value ± SD was recorded.

#### 4.6.6. Drug Content and pH Measurement

An accurately weighed amount of each gel formulation (0.5 gm) was dissolved in methyl alcohol to release AG, and then filtered using a membrane filter (0.2 μm, Whatman™Ltd., Maidstone, Kent, UK). The drug content in the filtrate was estimated spectrophotometrically at λ_max_ of 230 nm [69]. Since the prepared in situ gel formulations are intended for rectal administration, pH determination was required to ensure that the gel formulations are non-irritating. A calibrated pH meter (JENWAY 3310, Bibby Scientific Ltd., Felsted, Essex, UK) was used to measure the pH of the prepared in situ gel formulations in triplicate. The glass electrode of the equipment was dipped into the investigated in situ gels until a constant reading was recorded [12].

#### 4.6.7. In Vitro Drug Release Test

In vitro drug release was conducted using the USP paddle technique. The cellulose dialysis membrane was soaked overnight in distilled water before the experiment. An appropriate volume of the investigated in situ gel formulation equal to 10 mg AG was charged into the dialysis bag, which was then tied at both ends with cotton threads. The dialysis bag was then placed in a dissolution tester (SR II, 6 flasks, paddle type, Hanson Research Co., Chatsworth, CA, USA). The release media was 500 mL phosphate buffer, pH 7.4, heated at 37 ± 0.5 °C and stirred at a speed of 100 rpm. At specified time intervals for 12 h, 5 mL of the release medium was sampled and filtered. Every time a sample was withdrawn, the eliminated sample was promptly replenished with an equal volume of warmed fresh buffer medium to ensure a consistent volume (sink conditions) [70]. The filtrated samples were analyzed spectrophotometrically (double beam spectrophotometer, UV-1601, Shimadzu Co., Kyoto, Japan) at 230 nm after appropriate dilution with the fresh buffer as the blank [16]. The concentration of AG was estimated from a previously obtained standard curve. The experiments were performed in triplicate, and the results were the mean ± SD.

#### 4.6.8. Release-Data Analysis

For the determination of the appropriate kinetic model and the mechanism of the in vitro release of AG from the formulated in situ gels, the data of in vitro AG release were analyzed employing various mathematical models, namely zero, first, Higuchi-diffusion, Korsmeyer–Pappas, and Hixson–Crowell. The highest coefficient value (R^2^) referred to the order of AG release [71].

#### 4.6.9. Histopathology Study

The safety of the optimized in situ gel formula after rectal administration was investigated by the examination of male albino rabbit rectal specimens and observation of any histopathological changes [13]. Six male albino rabbits were divided into two groups (three rabbits each). They were fasted for 12 h before the experiment with free access to drinking water. Group I (untreated control group) was given saline, while group II received the optimized in situ gel formula rectally 5 cm above the anus using a plastic syringe at a dose of 5 mg/kg. After 24 h, the rabbits were killed, and the rectal tissues were removed, rinsed with saline solution, and placed in 5% *v*/*v* buffered formaldehyde. The fixed samples were dehydrated using ethyl alcohol 97% and fixed in paraffin. Rectal tissue sections of 4 µm thickness were stained with hematoxylin and eosin and inspected by a light microscope [72].

#### 4.6.10. Retention In Vivo Test

Methylene blue (as a dye) was added to the investigated in situ gel formulations at a concentration of 0.5% *w*/*w* to help in the observation of formulation leakage [41]. Six albino rabbits were used for the retention in vivo test. The optimized in situ gel formulation was administered at an AG dose of 5 mg/kg into the rectum 5 cm above the anus using a syringe with a gavage needle. Then, after waiting for 1 h, the staining around the anus of each rabbit was observed to determine whether the administered dosage form leakage occurred [16]. The number of rabbits with leakage was counted, and the leakage rate was calculated using the following equation.

$$\mathrm{Leakage}\;\mathrm{rate}=\frac{Number\;of\;rabbits\;with\;leakage}{Total\;number\;of\;rabbits}\times 100$$

#### 4.6.11. Pharmacokinetic Assessment

##### In Vivo Study

Nine male albino rabbits weighing 1.8–2.25 kg were randomly divided into three groups (I, II, and III) and housed individually in plastic cages at room temperature with free access to food and water. The rabbits were fasted for 24 h before and during the in vivo study. Group I (n = 3) received the optimized in situ gel formulation (5 mg/kg body weight) into the animal rectum 5 cm above the anus using a plastic syringe. Group II (n=3) received the unprocessed AG-loaded in situ gel formula with the same composition as the optimized formula (5 mg/kg body weight) into the animal rectum 5 cm above the anus using a plastic syringe. Group III (n = 3) received oral tablets of AG (5 mg/kg) by an intragastric tube (Agovald^®^ tablet). After administration of the different AG formulations, 1 mL blood samples were collected in a capped tube infiltrated with a heparin sodium solution at time intervals of 0.166, 0.5, 1, 1.5, 2, 3, 4, 6, and 8 h post-dosing from the marginal ear vein of each rabbit. Blood samples were centrifuged at 3000 rpm for 20 min. The obtained plasma samples were stored at −20 °C until analysis. For AG extraction, 500 μL of plasma samples were transferred to a labeled tube, then 2 mL ethyl acetate (extraction solvent) was added, and the mixture was vigorously shaken for 15 min and then centrifuged at 4000 rpm for 5 min. The separated supernatant was evaporated under nitrogen gas, and the residue was reconstituted with the mobile phase (100 µL) and filtered [73]. An Aliquot of 20 μL was injected into auto-sampler vials and assessed for the AG concentration by the HPLC method.

##### Pharmacokinetic Parameters (PK) Determination

The PK of AG was estimated utilizing the AG plasma concentration-time profile as illustrated in Table 8. The results obtained are expressed as mean ± SD of three determinations. The relative bioavailability (RB) of the optimized in situ rectal formulation against AG oral tablets in the rabbit plasma was estimated utilizing the following equation.

$$\mathrm{RB}=\frac{AUC0\text{–}\infty \;of\;optimized\;in\text{–}situ\;gel\;formulation}{AUC0\text{–}\infty \;of\;oral\;tablets}\times 100$$

#### 4.6.12. Accelerated Stability Study

The optimized in situ gel formula was subjected to an accelerated stability study to investigate the physical and chemical stability of the developed gel. The investigated samples were stored in screw-capped glass vials at various temperatures (25 ± 0.5 °C and 40 ± 1 °C/relative humidity 75% for 3 months [74]. Stored in situ gel formulation characteristics, such as color, pH, drug content, GT, GS, and in vitro drug release, were investigated at predetermined time intervals (1, 2, and 3 months). The experiments were conducted in a triplicate manner, and the data were expressed as the mean ± SD.

### 4.7. Statistical Analysis

Particle size, zeta potential, drug content, and cumulative % AG amount released at 6 h were presented as means ± SD and treated statistically using one-way analysis of variance (ANOVA). The statistical significance of differences between the pharmacokinetic parameters of different AG formulations was determined using one-way analysis of variance (ANOVA), and *p* < 0.05 was considered statistically significant.

## Figures and Tables

**Table 1:** The values of coefficients and ANOVA for dependent responses.

Source	Response								
	Gelation Temperature			Gel Strength			Cumulative Drug Release at 6 h		
	SOS	F-Value	*p*-Value	SOS	F-Value	*p*-Value	SOS	F-Value	*p*-Value
Model	103.08	24.84	0.002 *	516.33	2788.20	<0.0001 *	2138.25	141.75	<0.0001 *
A. P188%	88.17	63.73	0.0005	468.17	5056.20	<0.0001	1919.24	254.46	<0.0001
B. HPMC %	2.67	1.93	0.2237	48.17	520.20	<0.0001	219.01	29.04	0.0017
AB	12.25	8.86	0.0309						
Residual	6.92			0.5556			45.25		
Cor. Total	110.00			516.89			2183.50		

* Significant, SOS (Sum of Squares).

**Table 2:** Viscoelastic properties, syringeability, spreadability, drug content, and pH of the fabricated rectal in situ gel formulations.

F. Code	Viscosity, cP		Syringeability	Spreadability (cm^2^)	Drug Content, %	pH
	At 25 ± 1 °C	At 37 ± 0.5 °C				
F1	223 ± 14.57	18,425 ± 342.67	Pass	7.46 ± 0.31	90.18 ± 1.11	7.1 ± 0.46
F2	269 ± 10.89	20,841 ± 205.82	Pass	7.55 ± 0.22	93.83 ± 3.06	6.9 ± 0.65
F3	298 ± 15.04	26,279 ± 446.11	Pass	7.08 ± 0.18	89.67 ± 2.43	7.3 ± 0.31
F4	311 ± 14.72	28,560 ± 322.94	Pass	6.7 ± 0.35	94.25 ± 1.68	6.5 ± 0.11
F5	337 ± 11.82	31,807 ± 283.18	Pass	6.42 ± 0.19	92.41 ± 2.52	6.2 ± 0.37
F6	354 ± 12.33	34,572 ± 358.99	Pass	6.29 ± 0.37	95.78 ± 1.27	6.8 ± 0.76
F7	395 ± 10.19	35,226 ± 366.86	Pass	5.82 ± 0.29	94.26 ± 2.34	7.2 ± 0.61
F8	448 ± 9.37	37,005 ± 202.22	Pass	5.49 ± 0.11	96.55 ± 1.27	7.0 ± 0.25
F9	491 ± 12.74	39,204 ± 349.25	Pass	5.13 ± 0.24	93.02 ± 2.23	5.9 ± 0.43

**Table 3:** Kinetic analysis of the fabricated AG-AgNPs-loaded rectal in situ gel formulations.

F. Code	Zero-Order		First-Order		Diffusion		Korsmeyer		Hixson		Fitted Model
	R^2^	K_0_	R^2^	K_1_	R^2^	K_H_	R^2^	n	R^2^	K_C_	
F1	0.3957	3.6750	0.94292	0.18862	0.99074	41.287	0.95299	0.24927	0.22370	0.06244	Diffusion
F2	0.8927	7.8917	0.97323	0.13868	0.99987	29.391	0.98925	0.31955	0.98352	0.28842	Diffusion
F3	0.9025	6.5685	0.98974	0.10404	0.99240	26.718	0.98905	0.35188	0.97967	0.22695	Diffusion
F4	0.9163	6.2784	0.96155	0.07691	0.98768	25.290	0.96088	0.37446	0.97701	0.18772	Diffusion
F5	0.9381	6.0190	0.98928	0.05949	0.99288	23.805	0.99147	0.42423	0.97790	0.15814	Diffusion
F6	0.9429	5.9373	0.98318	0.05230	0.99516	23.322	0.99111	0.49985	0.97305	0.14515	Diffusion
F7	0.9625	5.8928	0.98922	0.04821	0.99597	22.672	0.99240	0.56105	0.98278	0.13750	Diffusion
F8	0.9693	5.6674	0.94191	0.04271	0.98892	21.606	0.93120	0.63590	0.98029	0.12548	Diffusion
F9	0.9742	5.3547	0.98949	0.03744	0.99363	20.203	0.99189	0.69238	0.98558	0.11305	Diffusion

**Table 4:** PK parameters of the optimized in situ gel, unprocessed AG-loaded in situ gel, and oral AG tablets and (mean ± SD, n = 3).

PK Abbreviation	Optimized In Situ Rectal Gel	Unprocessed AG-Loaded In Situ Gel	Oral AG Tablets
C_max_, ng/mL	1703.45 ± 35.62	1135.63 ± 40.34	734.29 ± 7.54
t_max_, h	2.5 ± 0.251	2.5 ± 0.189	3.5 ± 0.158
K_ab_, h^−1^	0.43945 ± 0.028	0.44063 ± 0.012	1.314 ± 0.073
K_el_, h^−1^	0.2712 ± 0.013	0.273279 ± 0.027	0.3234 ± 0.078
t_0.5ab_, h	1.576 ± 0.376	1.37273 ± 0.264	0.527 ± 0.251
t_0.5el_, h	2.56 ± 0.488	2.53 ± 0.367	2.142 ± 0360
AUC_0–8_, ng × h/mL	7068.262 ± 72.98	4692.648 ± 81.24	3039.735 ± 35.46
AUC_0–∞_, ng × h/mL	27,370.4 ± 250.26	18,036.65 ± 199.54	9860.745 ± 52.33
MAT, h	2.275 ± 0.150	2.269 ± 0.098	0.761 ± 0.012
AUMC, ng.h^2^/mL	34,659.45 ± 98.401	16,133.26 ± 103.44	14,175.89 ± 62.19
MRT, h	5.138 ± 0.035	4.148 ± 0.026	3.049 ± 0.0211
RB, %	277.5	182.9	-

**Table 5:** Accelerated stability study for the optimized rectal in situ gel formulation.

Parameter	Initial Values	At 25 ± 0.5 °C			At 40 ± 1 C°/ RH, 75%		
		Time of Sampling (Month)			Time of Sampling (Month)		
		1	2	3	1	2	3
Color	-	No change	No change	No change	No change	No change	No change
pH	7.20 ± 054	7.20 ± 0.21	7.20 ± 0.17	7.20 ± 0.56	7.20 ± 0.25	7.10 ± 0.72	7.00 ± 0.57
Drug content (%)	91.64 ± 4.1	91.53 ± 3.8	91.36 ± 3.5	91.10 ± 4.2	91.45 ± 3.7	90.72 ± 6.2	90.44 ± 3.8
GT (°C)	26.63 ± 2.1	26.99 ± 1.5	27.12 ± 3.2	27.79 ± 3.1	27.19 ± 4.6	27.58 ± 3.4	28.09 ± 1.9
GS (Sec)	36.98 ± 1.2	36.81 ± 1.7	36.75 ± 2.4	36.40 ± 1.9	36.7 ± 3.2	36.11 ± 1.8	35.67 ± 4.6
Rel. at 6 h (%)	80.24 ± 3.6	80.68 ± 2.8	81.39 ± 5.1	81.91 ± 4.7	81.05 ± 4.2	82.23 ± 3.9	83.25 ± 1.2

**Table 6:** Chromatographic conditions.

System	HPLC, JASCO Corporation, Tokyo, Japan)/PU–980 Pump, Autosampler Injector/Photodiode Detector (UV-Vis).
Column	Peerless LC–C18/RP, Altmann Analytik GmbH, Munich, Germany/250 mm (length)/4.6 mm (internal diameter)/5 μm (particle size).
Mobile phase	Methyl alcohol (HPLC grade): phosphate buffer, pH 3 (50:50 *v*/*v*).
Flow rate	1 mL/min.
Injection volume	20 μL.
Column temperature	25 °C.
Detection wavelength	230 nm.

**Table 7:** 3^2^ Factorial-design, independent variables, and the three responses of AG-AgNPs loaded rectal in situ gel formulations.

**Variable Code**	**Variable Name**		**Variable Level**			**Response Name**		**Constrains**
			**−1**	**0**	**+1**			
A	P188 percent (%, *w*/*v*)		10	15	20	Gelation temperature, °C		Minimization
						Gel strength, sec.		Maximization
B	HPMC K15M percent (%, *w*/*v*)		0.5	1	1.5	Cumulative percent drug released at 6 h, %		Maximization
**Run Code**	**NPs Equal to**	**P188** **(%, *w*/*v*)**	**HPMC K15M** **(%, *w*/*v*)**	**Na-Alg** **(%, *w*/*v*)**		**Y_1_ (°C) ± SD**	**Y_2_ (sec) ± SD**	**Y_3_ (%) ± SD**
F1	10 mg AG	10	0.5	0.5		30 ± 2.51	31 ± 1.11	98.67 ± 5.78
F2	10 mg AG	10	1	0.5		28 ± 1.68	33 ± 2.58	86.28 ± 1.46
F3	10 mg AG	10	1.5	0.5		26 ± 3.01	36 ± 1.69	81.98 ± 4.18
F4	10 mg AG	15	0.5	0.5		35 ± 2.69	39 ± 2.35	73.10 ± 4.58
F5	10 mg AG	15	1	0.5		33 ± 2.44	42 ± 2.70	67.69 ± 1.20
F6	10 mg AG	15	1.5	0.5		32 ± 3.21	45 ± 3.46	64.27 ± 2.34
F7	10 mg AG	20	0.5	0.5		34 ± 1.98	48 ± 3.57	59.13 ± 3.51
F8	10 mg AG	20	1	0.5		36 ± 3.27	51 ± 3.00	52.09 ± 2.30
F9	10 mg AG	20	1.5	0.5		37 ± 4.51	54 ± 1.58	48.40 ± 4.37

**Table 8:** Pharmacokinetics parameters.

PK Abbreviation	PK Full Name	Unit	Calculated from:
C_max_	Maximum plasma drug concentration	ng/mL	
t_max_	Time to achieve the C_max_	H	
K_ab_	Absorption rate constant	h^−1^	Plasma concentration- Time profile
K_el_	Elimination rate constant	h^−1^	
t_0.5ab_	Half-life of absorption	H	
t_0.5el_	Half-life of elimination	H	
AUC_0–8_	Areas under the plasma concentration– time curves from zero to the end of sampling time	ng.h/mL	The trapezoidal method
AUC_0–∞_	Areas under the plasma concentration–time curve from zero to infinity	ng.h/mL	AUC_0–8_ + last plasma concentration/K_el_
MAT	Mean absorption time	H	1/K_ab_
AUMC	Area under the first moment curve	ng.h^2^/mL	The linear trapezoidal rule with extrapolation to infinite time
MRT	Mean residence time	H	AUMC/AUC

## Data Availability

The datasets generated during and/or analyzed during the current investigation are available from the corresponding authors on reasonable request.

## References

[1] Yucel A.O., Demir-Dora D., İsbir M.F., Dora B. (2020). Agomelatine is superior to melatonin in pain suppression: An experimental study. Neurol. Sci. Neurophysiol..

[2] Saiz-Rodríguez M., Ochoa D., Belmonte C., Román M., Vieira de Lara D., Zubiaur P., Koller D., Mejía G., Abad-Santos F. (2019). Polymorphisms in CYP1A2, CYP2C9 and ABCB1 affect agomelatine pharmacokinetics. J. Psychopharmacol..

[3] De Berardis D., Fornaro M., Serroni N., Campanella D., Rapini G., Olivieri L., Srinivasan V., Iasevoli F., Tomasetti C., De Bartolomeis A. (2015). Agomelatine beyond borders: Current evidences of its efficacy in disorders other than major depression. Int. J. Mol. Sci..

[4] Ahmed S., Gull A., Alam M., Aqil M., Sultana Y. (2018). Ultrasonically tailored, chemically engineered and “QbD” enabled fabrication of agomelatine nanoemulsion; optimization, characterization, ex-vivo permeation and stability study. Ultrason. Sonochemistry.

[5] Shnoudeh A.J., Hamad I., Abdo R.W., Qadumii L., Jaber A.Y., Surchi H.S., Alkelany S.Z. (2019). Synthesis, characterization, and applications of metal nanoparticles. Biomaterials and Bionanotechnology.

[6] Kumar S., Kumar B., Sehgal R., Wani M., Kumar D., Sharma M.D., Singh V., Sehgal R., Kumar V. (2023). Advantages and disadvantages of metal nanoparticles. Nanoparticles Reinforced Metal Nanocomposites: Mechanical Performance and Durability.

[7] Nikolaidis P. (2020). Analysis of green methods to synthesize nanomaterials. Green Synthesis of Nanomaterials for Bioenergy Applications.

[8] Saddik M.S., Elsayed M.M., Abdelkader M.S.A., El-Mokhtar M.A., Abdel-Aleem J.A., Abu-Dief A.M., Al-Hakkani M.F., Farghaly H.S., Abou-Taleb H.A. (2021). Novel green biosynthesis of 5-fluorouracil chromium nanoparticles using harpullia pendula extract for treatment of colorectal cancer. Pharmaceutics.

[9] Gomes H.I., Martins C.S., Prior J.A. (2021). Silver nanoparticles as carriers of anticancer drugs for efficient target treatment of cancer cells. Nanomaterials.

[10] Laib I., Gheraissa N., Benaissa A., Benkhira L., Azzi M., Benaissa Y., Abdelaziz A.G., Tian F., Walsh M., Bechelany M. (2025). Tailoring innovative silver nanoparticles for modern medicine: The importance of size and shape control and functional modifications. Mater. Today Bio.

[11] Laghari S., Khuhawar M.Y. (2021). Rapid Visual Detection of Imipramine, Citalopram, and Sertraline by Citrate- Stabilized Silver Nanoparticles. Int. J. Nanosci. Nanotechnol..

[12] Sakran W., Abdel-Rashid R.S., Saleh F., Abdel-Monem R. (2022). Ethosomal gel for rectal transmucosal delivery of domperidone: Design of experiment, in vitro, and in vivo evaluation. Drug Deliv..

[13] Al-Joufi F., Elmowafy M., Alruwaili N.K., Alharbi K.S., Shalaby K., Alsharari S.D., Ali H.M. (2021). Mucoadhesive in situ rectal gel loaded with rifampicin: Strategy to improve bioavailability and alleviate liver toxicity. Pharmaceutics.

[14] Akl M.A., Ismael H.R., Abd Allah F.I., Kassem A.A., Samy A.M. (2019). Tolmetin sodium-loaded thermosensitive mucoadhesive liquid suppositories for rectal delivery; strategy to overcome oral delivery drawbacks. Drug Dev. Ind. Pharm..

[15] Salman Z., Alhamdany A., Yousif N. (2020). An innovative mucoadhesive thermosensitive in situ gelling liquid suppository of metoclopramide hydrocloride for treatment of nausea and vomiting associated with diseases. Indian J. Pharm. Sci..

[16] Yuan Y., Cui Y., Zhang L., Zhu H.-P., Guo Y.-S., Zhong B., Hu X., Zhang L., Wang X.-H., Chen L. (2012). Thermosensitive and mucoadhesive in situ gel based on poloxamer as new carrier for rectal administration of nimesulide. Int. J. Pharm..

[17] Rathi R., Sanshita, Kumar A., Vishvakarma V., Huanbutta K., Singh I., Sangnim T. (2022). Advancements in rectal drug delivery systems: Clinical trials, and patents perspective. Pharmaceutics.

[18] Zorraquín-Peña I., Cueva C., Bartolomé B., Moreno-Arribas M.V. (2020). Silver nanoparticles against foodborne bacteria. Effects at intestinal level and health limitations. Microorganisms.

[19] Vijayakumar A., Sharon E., Teena J., Nobil S., Nazeer I. (2014). A clinical study on drug-related problems associated with intravenous drug administration. J. Basic Clin. Pharm..

[20] Khan Z., Al-Thabaiti S.A., Obaid A.Y., Al-Youbi A.O. (2011). Preparation and characterization of silver nanoparticles by chemical reduction method. Colloids Surf. B Biointerfaces.

[21] Fahmy H.M., Mosleh A.M., Abd Elghany A., Shams-Eldin E., Serea E.S.A., Ali S.A., Shalan A.E. (2019). Coated silver nanoparticles: Synthesis, cytotoxicity, and optical properties. RSC Adv..

[22] Bélteky P., Rónavári A., Igaz N., Szerencsés B., Tóth I.Y., Pfeiffer I., Kiricsi M., Kónya Z. (2019). Silver nanoparticles: Aggregation behavior in biorelevant conditions and its impact on biological activity. Int. J. Nanomed..

[23] Mishra S.K., Teotia A.K., Kumar A., Kannan S. (2017). Mechanically tuned nanocomposite coating on titanium metal with integrated properties of biofilm inhibition, cell proliferation, and sustained drug delivery. Nanomed. Nanotechnol. Biol. Med..

[24] Pryshchepa O., Pomastowski P., Buszewski B. (2020). Silver nanoparticles: Synthesis, investigation techniques, and properties. Adv. Colloid Interface Sci..

[25] Kamble S., Agrawal S., Cherumukkil S., Sharma V., Jasra R.V., Munshi P. (2022). Revisiting zeta potential, the key feature of interfacial phenomena, with applications and recent advancements. ChemistrySelect.

[26] Ali M.H., Azad M.A.K., Khan K., Rahman M.O., Chakma U., Kumer A. (2023). Analysis of crystallographic structures and properties of silver nanoparticles synthesized using PKL extract and nanoscale characterization techniques. ACS Omega.

[27] Gelen V., Özkanlar S., Kara A., Yeşildağ A. (2024). Citrate-coated silver nanoparticles loaded with agomelatine provide neuronal therapy in acute cerebral ischemia/reperfusion of rats by inhibiting the oxidative stress, endoplasmic reticulum stress, and P2X7 receptor-mediated inflammasome. Environ. Toxicol..

[28] Liao Y., Zhang X., Li C., Huang Y., Lei M., Yan M., Zhou Y., Zhao C. (2016). Inclusion complexes of HP-β-cyclodextrin with agomelatine: Preparation, characterization, mechanism study and in vivo evaluation. Carbohydr. Polym..

[29] Fouda A., Abdel-Maksoud G., Saad H.A., Gobouri A.A., Mohammedsaleh Z.M., Abdel-Haleem El-Sadany M. (2021). The efficacy of silver nitrate (AgNO3) as a coating agent to protect paper against high deteriorating microbes. Catalysts.

[30] Ganguly S., Das P., Srinivasan S., Rajabzadeh A.R., Tang X.S., Margel S. (2024). Superparamagnetic amine-functionalized maghemite nanoparticles as a thixotropy promoter for hydrogels and magnetic field-driven diffusion-controlled drug release. ACS Appl. Nano Mater..

[31] Dong C., Zhang X., Cai H., Cao C. (2014). Facile and one-step synthesis of monodisperse silver nanoparticles using gum acacia in aqueous solution. J. Mol. Liq..

[32] Umer A., Naveed S., Ramzan N., Rafique M.S., Imran M. (2014). A green method for the synthesis of copper nanoparticles using L-ascorbic acid. Matéria.

[33] Indana M.K., Gangapuram B.R., Dadigala R., Bandi R., Guttena V. (2016). A novel green synthesis and characterization of silver nanoparticles using gum tragacanth and evaluation of their potential catalytic reduction activities with methylene blue and Congo red dyes. J. Anal. Sci. Technol..

[34] Ansari M.J., Rehman N.U., Ibnouf E., Alalaiwe A., Ganaie M.A., Zafar A. (2022). Gum acacia-and gum tragacanth-coated silver nanoparticles: Synthesis, physiological stability, in-vitro, ex-vivo and in-vivo activity evaluations. Coatings.

[35] Kayed K., Issa M., Al-Ourabi H. (2024). The FTIR spectra of Ag/Ag2O composites doped with silver nanoparticles. J. Exp. Nanosci..

[36] Bialik M., Kuras M., Sobczak M., Oledzka E. (2021). Achievements in thermosensitive gelling systems for rectal administration. Int. J. Mol. Sci..

[37] Fathalla Z.M., Vangala A., Longman M., Khaled K.A., Hussein A.K., El-Garhy O.H., Alany R.G. (2017). Poloxamer-based thermoresponsive ketorolac tromethamine in situ gel preparations: Design, characterisation, toxicity and transcorneal permeation studies. Eur. J. Pharm. Biopharm..

[38] Barse R., Kokare C., Tagalpallewar A. (2016). Influence of hydroxypropylmethylcellulose and poloxamer composite on developed ophthalmic in situ gel: Ex vivo and in vivo characterization. J. Drug Deliv. Sci. Technol..

[39] Agrawal M., Saraf S., Saraf S., Dubey S.K., Puri A., Gupta U., Kesharwani P., Ravichandiran V., Kumar P., Naidu V. (2020). Stimuli-responsive In situ gelling system for nose-to-brain drug delivery. J. Control. Release.

[40] Sonowal M.B. (2017). Formulation, Optimization and Evaluation of Novel Injectable, Thermoresponsive and Cytocompatible Gel for Sustained Drug Delivery. Int. J. Chemtech Res..

[41] Salem H.F., Ali A.A., Rabea Y.K., El-Ela F.I.A., Khallaf R.A. (2022). Glycerosomal thermosensitive in situ gel of duloxetine HCl as a novel nanoplatform for rectal delivery: In vitro optimization and in vivo appraisal. Drug Deliv. Transl. Res..

[42] Chen X., Zhang Y., Yu W., Zhang W., Tang H., Yuan W.-E. (2023). In situ forming ROS-scavenging hybrid hydrogel loaded with polydopamine-modified fullerene nanocomposites for promoting skin wound healing. J. Nanobiotechnol..

[43] Yurtdaş-Kırımlıoğlu G. (2022). A promising approach to design thermosensitive in situ gel based on solid dispersions of desloratadine with Kolliphor® 188 and Pluronic® F127. J. Therm. Anal. Calorim..

[44] Zhang X., Yu W., Zhang Y., Zhang W., Wang J., Gu M., Cheng S., Ren G., Zhao B., Yuan W.-E. (2024). A hydrogen generator composed of poly (lactic-co-glycolic acid) nanofibre membrane loaded iron nanoparticles for infectious diabetic wound repair. J. Colloid Interface Sci..

[45] Lee S.C., Gillispie G., Prim P., Lee S.J. (2020). Physical and chemical factors influencing the printability of hydrogel-based extrusion bioinks. Chem. Rev..

[46] Dewan M., Sarkar G., Bhowmik M., Das B., Chattoapadhyay A.K., Rana D., Chattopadhyay D. (2017). Effect of gellan gum on the thermogelation property and drug release profile of Poloxamer 407 based ophthalmic formulation. Int. J. Biol. Macromol..

[47] Abourehab M.A., Rajendran R.R., Singh A., Pramanik S., Shrivastav P., Ansari M.J., Manne R., Amaral L.S., Deepak A. (2022). Alginate as a promising biopolymer in drug delivery and wound healing: A review of the state-of-the-art. Int. J. Mol. Sci..

[48] Andrews G.P., Laverty T.P., Jones D.S. (2009). Mucoadhesive polymeric platforms for controlled drug delivery. Eur. J. Pharm. Biopharm..

[49] Dhawan S., Medhi B., Chopra S. (2009). Formulation and evaluation of diltiazem hydrochloride gels for the treatment of anal fissures. Sci. Pharm..

[50] Jackson T.C., Patani B.O., Ifekpolugo N.L., Udofa E.M., Obiakor N.M. (2019). Developent of metronidazole loaded silver nanoparticles from Acalypha ciliata for treatment of susceptible pathogens. Nanosci. Nanotechnol..

[51] Ashe B. (2011). A Detail investigation to observe the effect of zinc oxide and Silver nanoparticles in biological system. Biotechnology and Medical Engineering.

[52] Salunke B.K., Sathiyamoorthi E., Tran T.K., Kim B.S. (2017). Phyto-synthesized silver nanoparticles for biological applications. Korean J. Chem. Eng..

[53] Aziz S.B., Hussein G., Brza M., Mohammed S.J., Abdulwahid R., Saeed S.R., Hassanzadeh A. (2019). Fabrication of interconnected plasmonic spherical silver nanoparticles with enhanced localized surface plasmon resonance (LSPR) peaks using quince leaf extract solution. Nanomaterials.

[54] Elsayed M.M., Elsayed A., Fouad M.A., Mohamed M.S., Lee S., Mahmoud R.A., Sabry S.A., Ghoneim M.M., Hassan A.H., Abd Elkarim R.A. (2024). Development and optimization of vildagliptin solid lipid nanoparticles loaded ocuserts for controlled ocular delivery: A promising approach towards treating diabetic retinopathy. Int. J. Pharm. X.

[55] El-Shenawy A.A., Elsayed M.M., Atwa G.M., Abourehab M.A., Mohamed M.S., Ghoneim M.M., Mahmoud R.A., Sabry S.A., Anwar W., El-Sherbiny M. (2023). Anti-tumor activity of orally administered gefitinib-loaded nanosized cubosomes against colon cancer. Pharmaceutics.

[56] Elsayed M.M., Okda T.M., Atwa G.M., Omran G.A., Abd Elbaky A.E., Ramadan A.E.h. (2021). Design and optimization of orally administered luteolin nanoethosomes to enhance its anti-tumor activity against hepatocellular carcinoma. Pharmaceutics.

[57] Sabry S., Okda T., Hasan A. (2021). Formulation, characterization, and evaluation of the anti-tumor activity of nanosized galangin loaded niosomes on chemically induced hepatocellular carcinoma in rats. J. Drug Deliv. Sci. Technol..

[58] Hasan A.A., Sabry S.A., Abdallah M.H., El-Damasy D.A. (2016). Formulation and in vitro characterization of poly(dl-lactide-co-glycolide)/Eudragit RLPO or RS30D nanoparticles as an oral carrier of levofloxacin hemihydrate. Pharm Dev Technol.

[59] Zewail M., Gaafar P.M.E., Youssef N.A.H.A., Ali M.E., Ragab M.F., Kamal M.F., Noureldin M.H., Abbas H. (2023). Novel Siprulina platensis Bilosomes for Combating UVB Induced Skin Damage. Pharmaceuticals.

[60] Elsayed M.M., Aboelez M.O., Mohamed M.S., Mahmoud R.A., El-Shenawy A.A., Mahmoud E.A., Al-Karmalawy A.A., Santali E.Y., Alshehri S., Elsadek M.E.M. (2022). Tailoring of rosuvastatin calcium and atenolol bilayer tablets for the management of hyperlipidemia associated with hypertension: A preclinical study. Pharmaceutics.

[61] Hasan A., Abd Elghany M., Sabry S. (2020). Design and characterization of intra-oral fast dissolving tablets containing diacerein-solid dispersion. J. Appl. Pharm. Sci..

[62] Liu Y., Yang F., Feng L., Yang L., Chen L., Wei G., Lu W. (2017). In vivo retention of poloxamer-based in situ hydrogels for vaginal application in mouse and rat models. Acta Pharm. Sin. B.

[63] El-Kamel A., El-Khatib M. (2006). Thermally reversible in situ gelling carbamazepine liquid suppository. Drug Deliv..

[64] Raval M., Bagada H. (2021). Formulation and evaluation of cyclodextrin-based thermosensitive in situ gel of azithromycin for periodontal delivery. J. Pharm. Innov..

[65] Rençber S., Karavana S.Y., Şenyiğit Z.A., Eraç B., Limoncu M.H., Baloğlu E. (2017). Mucoadhesive in situ gel formulation for vaginal delivery of clotrimazole: Formulation, preparation, and in vitro/in vivo evaluation. Pharm. Dev. Technol..

[66] Shahien M.M., Alshammari A., Ibrahim S., Ahmed E.H., Atia H.A., Elariny H.A., Abdallah M.H. (2024). Development of Glycerosomal pH Triggered In Situ Gelling System to Ameliorate the Nasal Delivery of Sulpiride for Pediatric Psychosis. Gels.

[67] Okur N.Ü., Yozgatlı V., Şenyiğit Z. (2020). Formulation and detailed characterization of voriconazole loaded in situ gels for ocular application. J. Fac. Pharm. Ank. Univ..

[68] Abdallah M.H., Abdelnabi D.M., Elghamry H.A. (2022). Response Surface Methodology for Optimization of Buspirone Hydrochloride-Loaded In Situ Gel for Pediatric Anxiety. Gels.

[69] Fathi A.M., Eissa R.G., Balata G.F., Ghazy F.-E.S., Eissa N.G. (2023). Intranasal thermosensitive hydrogel of agomelatine solid dispersion for better management of depression. J. Drug Deliv. Sci. Technol..

[70] Abdallah M.H., Sabry S.A., Hasan A.A. (2016). Enhancing transdermal delivery of glimepiride via entrapment in proniosomal gel. J. Young Pharm..

[71] Barzegar-Jalali M. (2008). Kinetic analysis of drug release from nanoparticles. J. Pharm. Pharm. Sci..

[72] Fawaz F., Bonini F., Guyot M., Lagueny A., Fessi H., Devissaguet J. (1996). Disposition and protective effect against irritation after intravenous and rectal administration of indomethacin loaded nanocapsules to rabbits. Int. J. Pharm..

[73] Zhang H., Pu C., Wang Q., Tan X., Gou J., He H., Zhang Y., Yin T., Wang Y., Tang X. (2019). Physicochemical characterization and pharmacokinetics of agomelatine-loaded PLGA microspheres for intramuscular injection. Pharm. Res..

[74] Madhavi Harika S., Sudhakar M., Basava Rao V. (2022). Formulation and Characterization of Carvedilol In situ Gels for Oral Delivery-In vitro and In vivo Pharmacokinetic Studies. Anal. Chem. Lett..

